# De novo in vitro shoot morphogenesis from shoot tip-induced callus cultures of *Gymnema sylvestre* (Retz.) R.Br. ex Sm

**DOI:** 10.1186/s40659-019-0211-1

**Published:** 2019-01-19

**Authors:** Tasiu Isah

**Affiliations:** Department of Botany, School of Chemical and Life Sciences, Hamdard University, New Delhi, 110 062 India

**Keywords:** Plant tissue culture, Plant regeneration, Plant growth regulators, Phytochemicals, *Gymnema sylvestre*, Gymnemic acid, Micro shoots, Plantlets, Shoot morphogenesis

## Abstract

**Background:**

*Gymnema sylvestre* is a medicinal woody perennial vine known for its sweetening properties and anti-diabetic therapeutic uses in the modern and traditional medicines. Its over-exploitation for the therapeutic uses and to meet the demand of pharmaceutical industry in raw materials supply for the production of anti-diabetic drugs has led to considerable decline in its natural population.

**Results:**

An efficient system of shoot bud sprouting from nodal segment explants and indirect plant regeneration from apical meristem-induced callus cultures of *G. sylvestre* have been developed on Murashige and Skoog (MS) medium amended with concentrations of cytokinins. Of the three growth regulators tested, *N*^6^-benzylaminopurine (BAP) was the most efficient and 2.0 mg L^−1^ gave the best shoot formation efficiency. This was followed by thidiazuron (TDZ) and kinetin (Kin) but, most of the TDZ-induced micro shoots showed stunted growth. Multiple shoot formation was observed on medium amended with BAP or TDZ at higher concentrations. The produced micro shoots were rooted on half strength MS medium amended with auxins and rooted plantlets acclimatized with 87% survival of the regenerates.

**Conclusions:**

The developed regeneration system can be exploited for genetic transformation studies, particularly when aimed at producing its high yielding cell lines for the anti-diabetic phytochemicals. It also offers opportunities for exploring the expression of totipotency in the anti-diabetic perennial vine.

## Background

Phytomedicine is the greatest source for the remedy of diseases nowadays and over 80% of human population relies on it for health care delivery due to the minimal side effects and low cost [1–4]. The increase in number of diabetic patients around the globe has led to exploitation of natural plant sources for its remedy among which *Gymnema sylvestre* is highly exploited for the therapeutic use. The woody climber belongs to the family Asclepiadaceae and is recognized in the traditional systems of medicine for the remedy of diabetes mellitus and several other therapeutic uses [1]. Its leaves produce glycosides, anthraquinones and gymnemic acid, which are ascribed the antidiabetic, sweetening and anti-inflammatory properties [1, 5]. Overexploitation of *G. sylvestre* natural population coupled with poor seed germination capacity and low rooting ability during vegetative propagation are impediments to its rapid clonal multiplication to meet phytochemical demand of the pharmaceutical industry in raw materials supply for the production of anti-diabetic drugs and other medicinal uses [6].

In vitro clonal propagation offers rapid and efficient strategy for the production of disease-free clones *en masse* and for the conservation of natural population [7, 8]. It also offer opportunities for the large-scale production and isolation of bioactive molecules produced by *G. sylvestre* that are used in the industrial production of its anti-diabetic drugs. However, initiating the in vitro cultures is constrained by difficulties in appropriate explant availability in a season and obtaining the most suitable one for the in vitro clonal production. De novo shoot morphogenesis forms the basis for which new genetic variations and biotechnological approaches can be applied to improve clonal multiplication of medicinal plants *en masse*, particularly when mature tissues can be used [7–11]. Further, indirect regeneration can be used to study details of dedifferentiation events through the developmental stages, with application in genetic transformation studies and selection of superior clones [7, 12, 13]. Developing the in vitro clonal propagation strategy has application in the large-scale production of *G. sylvestre* for its clones of therapeutic uses such as in the treatment of diabetes, the sweetening properties, and other pharmaceutical uses. Factors that influence in vitro clonal propagation efficiency of the *G. sylvestre* includes seedling age, explant, basal medium, plant growth regulators (PGRs) types and concentrations, organic supplements, antioxidants added into the culture medium and physiological state of explants [6].

Previous in vitro clonal propagation of *G. sylvestre* were achieved from mature explants through direct regeneration pathways [6, 14–16] and somatic embryogenesis (SE) from seedling explants [17] but, totipotency expression potential of its cells via de novo organogenesis from meristematic tissues-derived callus cultures have not been studied. The de novo shoot morphogenic pathway is not attainable with many woody plants due to the influence of genotype and specific requirement for certain cells having unique properties needed for initiation of meristematic primordial essential for the in vitro morphogenesis [18, 19]. In the present study, axillary bud sprouting of nodal segment explants and plant regeneration from shoot tip-induced callus cultures were achieved in *G. sylvestre,* indicating totipotency expression potentials of the callus cultures.

## Results

**Fig. 1:**
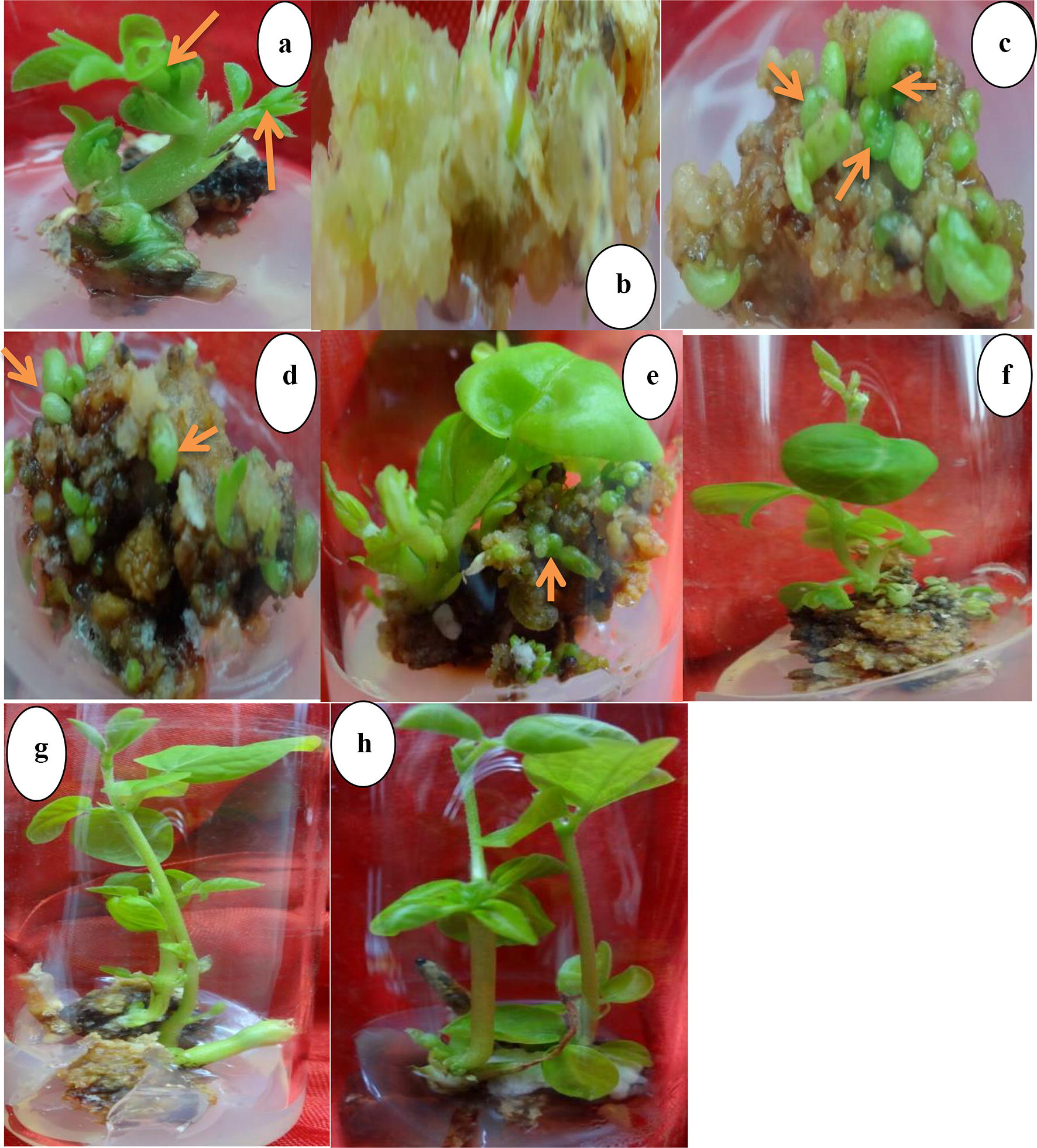
**a** Micro shoot bud produced on MS medium added with BAP (1.0 mg L^−1^) using ex vitro obtained nodal segment explant from which shoot-tip was excised for callus induction, **b** callus induced from the excised shoot-tip explants, **c**–**f** de novo shoot morphogenesis from the apical meristem-induced callus on MS medium amended with TDZ (1.0 mg L^−1^) after 5 weeks culture, **g**, **h** elongation of the induced micro shoots on medium added with BAP (1.0 mg L^−1^) after 8 weeks culture

**Fig. 2:**
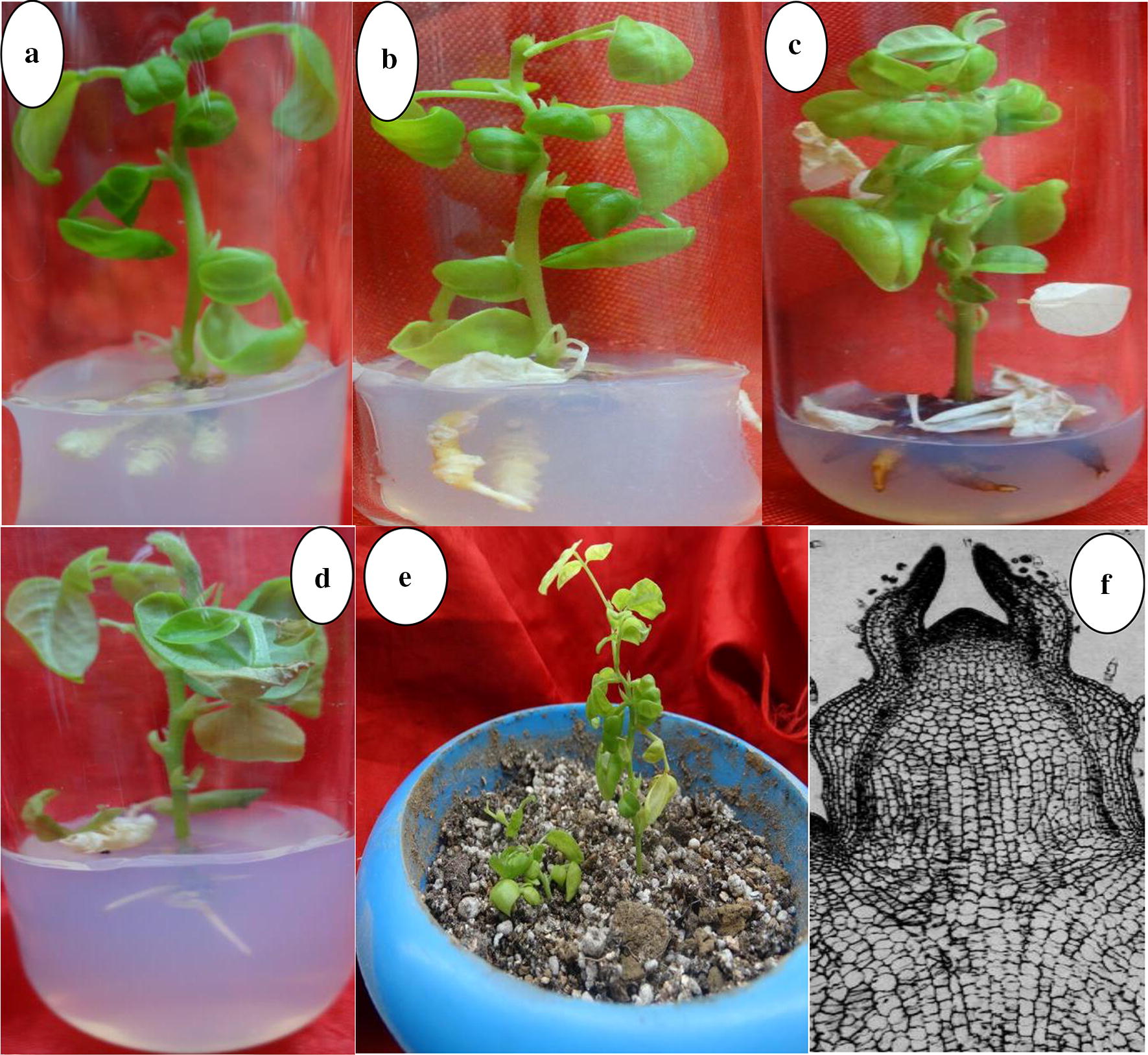
**a**–**d** Rhizogenesis of the obtained micro shoots on solid media added with auxins; **a** IAA (1.0 mg L^−1^), **b** NAA (2.0 mg L^−1^), **c** IBA (2.0 mg L^−1^), **d** IBA (1.0 mg L^−1^) after 3 weeks culture. **e** Acclimatization of the regenerated plants in a potted mixture of soilrite:perlite (1:1) after 2-months, **f** histological study of the micro shoots

Cultured ex vitro collected and surface sterilized *G. sylvestre* nodal segment explants produced shoot buds within 3–4 weeks culture on solid MS medium added with most of the cytokinin types and concentrations tested (Fig. 1a). The response was earlier and more in cultures this growth regulators were amended at higher levels (above 1.0 mg L^−1^). For the organogenesis, shoot bud apical meristem of the induced micro shoots were used to initiate callus cultures (Fig. 1a, b). When upper most apical meristem of young buds (about 1–3 mm) were excised and cultured on solid MS medium added with 2,4-dichloropenoxy acetic acid (2,4-D) (2.0 mg L^−1^), profuse callus formation was observed within 2–3 weeks culture (Fig. 1b). The induced callus was yellowish-brown and faster in biomass production. Subsequent subculture of the callus onto fresh solid medium added with the same PGR concentration resulted in its slow growth accompanied with darkening of the yellowish-brown calli and increased compactness. Calli were then cultured on medium added with selected cytokinin types and concentrations for the organogenesis study. The response of the callus cultures to shoot morphogenesis was dependent on PGRs type and concentration amended into the cultivation medium (Table 1). After 4–5 weeks culture, formation of shoot buds were observed in most of the cultures, and was at higher frequency on media added with PGRs at 1–4 mg L^−1^ while 0.5 mg L^−1^ showed less efficiency (Fig. 1c–f). Histological studies showed densely packed meristematic cells in the upper-most region of the induced micro shoot apices and larger parenchymatous cells in the lower (Fig. 2f).

**Table 1:** Effect of cytokinins on indirect shoot morphogenesis from apical meristem-induced callus cultures of *Gymnema sylvestre* cultivated for 6 weeks on solid Murashige and Skoog medium

Concentration of the cytokinins (mg L^−1^)					
BAP	TDZ	Kin	Number of shoots per ~ 0.5 g calli mass	Shoot buds initiated (%)	Length of the micro shoots in cm (mean ± SE)
0.5	–	–	3.94 ± 0.42d	41.16 ± 3.11c	1.34 ± 0.62d
1.0	–	–	6.87 ± 0.97a	63.42 ± 2.81a	2.17 ± 0.55b
2.0	–	–	7.92 ± 0.81a	79.42 ± 2.73a	2.38 ± 0.43a
3.0	–	–	5.39 ± 0.77b	58.4 ± 3.17b	2.24 ± 0.39a
4.0	–	–	4.03 ± 0.91c	49.81 ± 2.16c	2.14 ± 0.32b
	0.5	–	2.87 ± 0.34e	38.93 ± 2.18d	1.18 ± 0.48c
	1.0	–	5.43 ± 0.42b	58.37 ± 1.62b	2.01 ± 0.32b
	2.0	–	6.78 ± 0.67a	63.41 ± 2.44a	2.14 ± 0.41b
	3.0	–	4.93 ± 0.51c	51.22 ± 3.81b	2.02 ± 0.36b
	4.0	–	3.67 ± 0.47d	42.81 ± 2.94c	1.99 ± 0.29c
		0.5	2.14 ± 0.25e	31.42 ± 1.15d	1.02 ± 0.22e
		1.0	4.21 ± 0.33c	47.34 ± 1.26c	1.86 ± 0.34c
		2.0	5.17 ± 0.18b	54.49 ± 2.21b	2.02 ± 0.29b
		3.0	3.21 ± 0.26d	39.27 ± 2.45d	1.78 ± 0.41c
		4.0	2.11 ± 0.34e	31.38 ± 1.87d	1.56 ± 0.28d

Obtained experimental data were inverse transformed prior to the statistical analysis. Mean values within column that are followed by the same letter are not significantly different at p < 0.05 levels

**Fig. 3:**
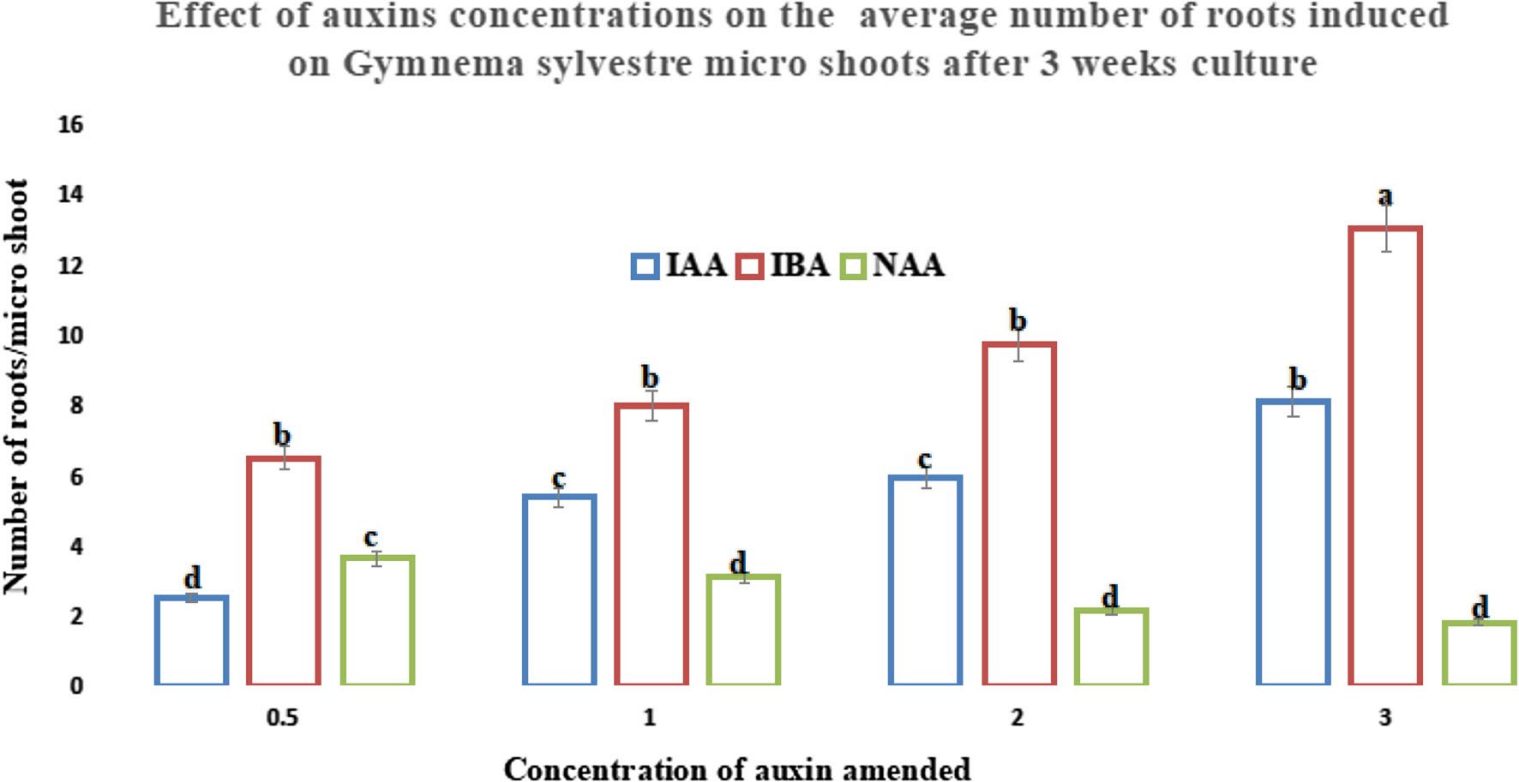
Results of micro shoots rhizogenesis on media added with auxins at different concentrations

In this study, maximum shoot formation was observed with *N*^6^-benzylaminopurine (BAP) added media followed by thidiazuron (TDZ) and least with the kinetin (Kin) (Table 1). Of the five concentrations tested, BAP at 2.0 mg L^−1^ was the most efficient; it produced an average of 7.92 ± 0.81 micro shoots with an average length of 2.38 ± 0.43 cm per approximately 0.5 g callus at 79% efficiency while Kin at 0.5 mg L^−1^ was the least effective (Table 1). The micro shoots cultivated through three subcultures were separately excised for rooting using half strength MS medium added with indole 3-acetic acid (IAA), indole 3-butyric acid (IBA) or naphthalene acetic acid (NAA) concentrations (0.5–3 mg L^−1^). In most of the cultured micro shoots, root formation occurred within 3 weeks culture, and the observed rooting response evaluated and presented in Fig. 3, with a good sample in Fig. 2d. Higher concentration of IBA (above 1.0 mg L^−1^) produced large but longer, thick and abnormal-looking roots (Fig. 2c) while NAA and IAA at high and low levels were inefficient for rooting the micro shoots (Fig. 2a, b). However, best formation of the roots was achieved with the IBA-added media in comparison to the other auxins tested (Fig. 3).

## Discussions

### Shoot formation

The establishment of in vitro cultures for the clonal multiplication of medicinal plants offers an alternative strategy for the conservation and production of phytochemicals they produce. Factors that influence the in vitro morphogenesis includes explant type and its physiological ontogenetic age, the season of collection and size, among others [7, 20]. In the case of *G. sylvestre* and many species, the in vitro morphogenesis could only be achieved when explants used to initiate the in vitro cultures are at a certain stage of development [6, 17, 19]. In previous studies, the indirect plant regeneration system of *G. sylvestre* were attempted using mature explant tissues of leaf, hypocotyl, cotyledons and nodal segment explants [17, 21, 22]. This prompted the experiment reported herein using ex vitro nodal segment of mature plants as an initial explant used to initiate micro shoot and from which the plant regeneration system was developed using micro shoot apical meristem. The juvenility of shoot-tip explant and its usual high IAA levels made it an ideal system for initiating callus cultures and subsequent organogenesis studies, and a possible explanation to the profuse callus induction observed in this study. Further, the advantages of developing indirect plant regeneration system of *G. sylvestre* using the apical meristem could have application in the production of disease-free plants which had been achieved with many woody and medicinal plants such as strawberry [23], *Jatropha curcas* [24] and recently with the Chilean strawberry [25]. For the organogenesis, calli were cultured on MS medium added with cytokinins at different concentrations due differential role they play in promoting cell division, the other aspects of in vitro morphogenesis that includes plant growth physiology and development by regulating gene expression, such as the expression of knotted 1 (kn1) homeobox family (exclusively expressed in apical meristem) essential for the in vitro morphogenesis [26]. The shoot-tip induced callus cultured on MS medium augmented with the cytokinins produced shoot buds at varied degree (by concentration of the cytokinins amended) within 2 weeks culture while for the control callus cultivation, proliferation without shoot bud initiation were observed (Fig. 1b–e). With the increased culture duration, the number, length and percentage micro shoot formation per culture tube showed variations associated with the cytokinins amended in the culture medium. Of the three cytokinins tested, BAP was the most efficient, followed by TDZ and Kin. However, higher concentrations of the cytokinins proved inhibitory to morphogenesis parameters (Table 1), which explains differential cellular effect of the cytokinins amended in the culture medium. The cytokinins are essential compositions of plant tissue culture media that influence in vitro morphogenesis by their types and concentration(s) amended in the cultivation medium, and show variant effects on shoot growth parameters due to differential influence on growth and physiology through accumulation as endogenous cytokinins determined by rate of cellular metabolism. Previous studies on the organogenesis capacity of *G. sylvestre* on medium supplemented with BAP encountered formation of green, compact and hard granular callus when leaf and nodal segments were used as initial explants [21]. Kin produced light green, less hard and compact callus with greater biomass production. Combination of BAP with auxins produced more callus biomass over that with the Kin. Culture of this calli in liquid medium further enhanced the responses without shoot morphogenesis. Khatak et al. [22] obtained green callus cultures from leaf explants of *G. sylvestre* and pigmentation of the callus increased with increase in the concentration of cytokinins amended in the cultivation medium and subculture cycles, without morphogenesis. However, Ashok Kumar et al. [17] have earlier obtained embryogenic callus and subsequent SE when hypocotyl, leaf and cotyledons of *G. sylvestre* seedlings were used as initial explants for establishment of its in vitro cultures. In the meristem culture and subsequent micropropagation of Chilean strawberry accessions, the use of BAP in the cultivation medium increased plantlets multiplication up to 3–6 micro shoots/explant [25] while it gave more number of shoots over Kin when amended in the culture medium of *Senna occidentalis* hypocotyl-derived callus cultures [12]. In a recent reported experience with the in vitro cultures of *Chonemorpha fragrance*, TDZ showed more efficiency over BAP and Kin in the induction and proliferation of micro shoots [27]. In this experiment, compared to the other cytokinins tested, most of the TDZ-induced micro shoots showed stunted growth and were relatively shorter when compared to those produced by BAP-amended medium cultures. However, their subsequent culture on BAP, Kin-added or PGRs-free media promoted elongation, suggesting the inhibitory effect of TDZ on micro shoots elongation (Fig. 1g, h). Further, subculture of the micro shoots at 3–4 weeks interval per round to the fresh solid medium as the induction medium promoted high frequency multiple shoot formation by third subculture cycle. Formation of the multiple shoots was more on solid medium cultivation of the cultures added with higher levels of BAP or TDZ. Multiple shoot formation in the in vitro cultures of *G. sylvestre* had earlier been reported [6, 16] as well as with medicinal plants such as *Mucuna pruriens* [28] and *Isolon wightii* [29]. In the present experiment, multiple shoot formation was not observed on medium supplemented with Kin at higher and lower concentrations. In the shoot tip-induced callus cultures of *Capsicum chinense* Jacq. cv. Naga King Chili [30] and inverted hypocotyl explant-induced callus cultures of *Solanum melongena* L. cv. Arka, callus proliferation and pigmentation was accompanied by the induction of micro shoot primordial with TDZ as the most effective PGR among the others tested [30, 31]. In most of the cultures used in the experiment reported herein, medium browning due to the leaching effect of phenolic compounds by callus and the produced micro shoots were observed. To minimize the effect, addition of ascorbic acid at 0.5 mg L^−1^ was attempted with success.

### Root formation

Rhizogenesis is an essential stage but difficult task with most woody plants due to their differential response to root initiation in the in vitro cultures as well as during conventional methods of clonal propagation. For many of their in vitro cultures, addition of media supplements have become a requirement for stimulating rhizogenesis, due to the differential response they show to auxins metabolism which is influenced by the in vitro culture physiology. This is in turn determined by the medium composition for which the shoots were initiated in addition to genetic factors. In the present experiment, root induction from micro shoots was observed after 2 weeks cultivation in most of the cultures auxins were amended in the culture medium. Better formation of thin and healthy roots was achieved on half strength MS medium added with IBA at 1.0 mg L^−1^ (Fig. 2d). The induced roots elongated with lateral root formation from the primary roots, with increase in the culture duration using most IBA-amended medium cultures. In the case of NAA and IAA, abnormal root formation was observed in many cultures they were amended above 1.0 mg L^−1^ (Figs. 1, 2). Control cultures and those cultivated on media added with higher levels of NAA showed basal callusing of the micro shoots. Compared to the other auxins tested for the rhizogenesis of *Capsicum chinense* Jacq. cv. and *Solanum melongena* cv. Arka Shirish micro shoots, IBA was found more efficient over other auxins tested [30, 31] while higher levels of NAA resulted in abnormal and thick root formation in *S. occidentalis* [12]. In the present experiment, the in vitro regenerated plants were acclimatized in potted mixture of soilrite:perlite (1:2) and covered with polyethylene bags to retain high relative humidity in a controlled environment for 2 weeks. Later, the polyethylene bags were removed (Fig. 2e) for acclimatization of plantlets in green house before their transfer into the field condition for which 87% survival of regenerated plants were recorded after 2 months.

## Conclusions

In conclusion, an efficient indirect plant regeneration system from callus cultures induced from micro shoot apical meristem that were in turn derived from ex vitro nodal segment explants of *G. sylvestre* have been developed. The system can be exploited for genetic transformation studies, particularly when aimed at producing its anti-diabetic phytochemicals through production of high yielding cell lines. It also offers opportunities for exploring the expression of totipotency in the callus cultures of the anti-diabetic medicinal woody vine.

## Methods

### Explant collection, preparation and cultures establishment

Healthy nodal segment explants (from actively growing twigs) of about 1.0–2.0 cm length were collected from about 5-years-old *G. sylvestre* (during early morning hours) grown in the herbal garden of Botany department, Hamdard University New Delhi, India (28° 33′ 41.9652″ N and 77° 16′ 52.5288″ E) during the months of October and November, 2013. The explants were washed thoroughly (using a pinch of cetrimide dissolved in tap water) for 1–3 min before subjected to the jet of running tap water for 10–20 min. Surface sterilization of the explants was performed in laminar air hood chamber using 70% ethanol treatment for 2–5 min followed by 2–3 rinses with sterile Milli-Q water. Further surface sterilization was done using freshly prepared 0.1% mercuric chloride solution for 2–3 min followed by rinses with sterilized Milli-Q water 2–3 times. The explants (about 1.0–2.0 cm length) were allowed to partially dry up adherent water (in petri plate) and the basal region excised prior to the culture on MS medium [32] added with PGRs. Induced micro shoot bud (apical meristem of about 1–3 mm in height) were excised and used for callus induction (Fig. 1a, b) on solid MS medium supplemented with 2,4-D (2.0 mg L^−1^). After 6 weeks culture, the produced calli were cultured onto fresh medium added with the same auxin concentration (two subculture cycles). Proliferated calli were then cultured on medium added with concentrations of BAP, Kin or TDZ at 0.5, 1.0, 2.0, 3.0 or 4.0 mg L^−1^ for the de novo shoot organogenesis studies. After 6 weeks culture, the number of shoots formed/culture tube, their length, and percentage formation were evaluated, and obtained data analyzed. The micro shoots were cultivated for three subcultures on media containing same PGRs as the induction medium before separately excised for rooting on half strength MS medium added with IAA, IBA or NAA concentrations (0.5–3 mg L^−1^) for rhizogenesis. The micro shoots were cultivated on solid half strength MS medium added with the auxins concentrations essential for root formation, and experimental data collected after 3 weeks culture analyzed.

In this experiment, the MS medium used contained 3% sucrose and media pH adjusted to 5.6–5.8 using 1 N NaOH or HCl. The media were solidified with 8% Agar (Agar Agar Microbiology Mumbai, India) before autoclaved at 121 °C for 21 min. All cultures were maintained under 12 h day/light photoperiod provided by cool fluorescent tubes (Phillips India) having photon flux density of 40 W, 50 μmol m^−2^ s^−1^ and culture room temperatures of 25 ± 2 °C.

### Histology

For the histological studies, upper most regions of the induced micro shoots (about 1–3 mm) were fixed in formalin–acetic acid–alcohol (FAA) (95%) that contained ethanol: glacial acetic acid: formaldehyde: water (10:1:2:7). This was followed by dehydration in graded series of ethanol (20, 30, 50, 70, 90, and 100%) for 20 min at each of the steps through three changes. The fixed tissues obtained were embedded in paraffin wax before cut into thin sections (10 µm) using microtome. Sectioned strips were stained with hematoxylin and eosin followed by gentle washing with Milli-Q water and then dried at 42 °C. The strips were further washed using xylol and mounted on clean glass slides using DPX followed by visual observation under a microscope (Leica, Germany).

### Statistical analysis

The experiments were carried out in triplicate sets of eight replicates per treatment and the obtained data analyzed using SPSS ver. 21 (USA). Significant differences between the treatments was assessed by analysis of variance (ANOVA) followed by Tukey’s range test at p < 0.05 and the obtained data presented as mean ± standard error of the triplicate sets of experiments.

## Abbreviations

MS: Murashige and Skoog medium
BAP: *N*^6^-benzylaminopurine
TDZ: thidiazuron
Kin: kinetin
NAA: naphthalene acetic acid
IAA: indole 3-acetic acid
IBA: indole 3-butyric acid
2,4-D: 2,4-dichlorophenoxy acetic acid
